# Using magnetic resonance imaging to quantify the inflammatory response following allergen challenge in allergic rhinitis

**DOI:** 10.1002/iid3.86

**Published:** 2015-09-17

**Authors:** Brian R. Leaker, Glenis Scadding, C. Richard Jones, Peter J. Barnes

**Affiliations:** ^1^ Respiratory Clinical Trials LtdLondonW1G 8HUUK; ^2^ Royal National ThroatNose and Ear HospitalLondonWC1X 8DAUK; ^3^ National Heart and Lung InstituteImperial CollegeLondonSW3 6LYUK

**Keywords:** Acoustic rhinometry, allergic rhinitis, MRI, nasal allergen challenge

## Abstract

Current rhinometric and flow assessments measure nasal patency and are often poorly correlated with rhinitis symptoms. To evaluate magnetic resonance imaging (MRI) as a new method to measure inflammatory changes in nasal and sinus mucosa following nasal allergen challenge. A pilot study (*n* = 6) determined the optimal technical settings for MRI to measure inflammatory change which were then adopted for the main study. This study was a single blind, placebo‐controlled, three‐way crossover trial in 14 subjects with seasonal allergic rhinitis. Effects of cetirizine, cetirizine and pseudoephedrine (Cet+PE), or placebo on total nasal symptom scores (TNSS), peak nasal inspiratory flow (PNIF), nasal nitric oxide (nNO), acoustic rhinometry, and MRI end points following nasal intranasal allergen challenge were measured. There were significant changes in all parameters after allergen challenge (*P *< 0.01), except for nNO. MRI end points were less variable and more consistent than PNIF and acoustic rhinometry in detecting changes after allergen challenge. Total nasal airspace volume was the most sensitive and reproducible MRI measurement, with a mean reduction from −5.37 cm^3^ (95%CI −7.35, −3.38; *P *< 0.001), which was maximal 60 min after allergen challenge. A change of one in TNSS corresponded to a change in MRI volume of −0.57 cm^3^. There was an improvement in all parameters (except nNO) in subjects taking Cet+PE compared with placebo, however this did not achieve significance probably because of the small study size (overall analysis *P *> 0.07; comparison of active versus placebo *P *> 0.09). MRI provides novel insights into the anatomical inflammatory changes post allergen challenge and provides a new method for assessment of nasal patency and objective measurement of inflammatory responses.

**Abbreviations**
ARDX: Acoustic rhinometry distance of minimal cross sectional area
ARMXCA: Acoustic rhinometry of nasal volume and minimum cross sectional area
ARV: Acoustic rhinometery volume
ARX: Acoustic rhinometry cross sectional area
METASV: Average airspace sectional area
MIXA: Minimum cross sectional area
MRI: Magnetic resonance imaging
NCV: Nasal cavity volume
nNO: Nasal nitric oxide
NSSC: Nasal symptom score for congestion
NTV: Nasal tissue volume
PD: Proton density
PNIF: Peak nasal inspiratory flow
PNIFMAX: Maximum peak nasal inspiratory flow
TASV: Total airspace volume
TMSA: Total mucosal surface area
TNCV: Total nasal cavity volume
TNCVm: Normalized TNCV
TNSS: Total nasal symptom score
TNTV: Total nasal tissue volume
T2: Transverse relaxation time
VAS: Visual analog scale
VASCR: Visual analog scale score

## Introduction

Nasal provocation has been used to investigate the pathophysiology of allergic and non‐allergic rhinitis, and is thought to have potential value for the evaluation of mechanisms of inflammation in both upper and lower airways, because of the similarity of the inflammatory responses to allergen challenge 1. Traditionally, the evaluation of therapies for allergic rhinitis have used symptom‐based scores (total nasal symptom score; TNSS) or visual analog scale (VAS) methods, despite the subjective nature and high variability of these tests 2, 3, 4, 5. There is a need for more objective and reproducible biomarkers of allergic inflammation which are non‐invasive to facilitate the clinical assessment of novel anti‐inflammatory drugs.

H_1_‐receptor antagonists (anti‐histamines) are widely prescribed for the treatment of allergic rhinitis but have limited clinical efficacy. Anti‐histamines may relieve symptoms of itch, sneeze, and discharge but are less effective treatments for nasal blockage. Nasal blockage may result from oedema and vascular congestion. Therefore, the combination of the antihistamine cetirizine (Cet) 6 and a decongestant, such as pseudoephedrine (PE), has a logical rationale 7, 8. We compared the effect of single doses of Cet (10 mg) alone or Cet (10 mg) and PE (120 mg) in combination versus placebo to evaluate MRI end points against symptom scores (TNSS, VAS, and nasal symptom score for congestion NSSC), measurements of nasal patency 4, 9, acoustic rhinometry, peak nasal inspiratory flow (PNIF), acoustic rhinometry of nasal volume and minimum cross sectional area (ARMXCA) and nasal nitric oxide (nNO) 10, 11, 12. The study was conducted in two parts, with an initial pilot phase to determine the relevant MRI parameters and a second study as a single‐blind three‐way cross over study.

## Methods

### Subjects

Asymptomatic subjects with a diagnosis of seasonal allergic rhinitis and a previous positive skin prick test for Timothy grass pollen at or within the 12 months preceding the screening visit were selected. Six subjects were recruited to part 1 of the study. Fourteen subjects were randomized in part 2, this included the six subjects from part 1. All subjects were included in the analysis, the average age of subjects was 31 years with a male to female ratio of 12:2. Full details of the subject characteristics at baseline can be found in the supplemental data online (Table SI). This prospective study was approved by the East London and the City Research Ethics Committee (07/Q0603/3) and written informed consent was obtained from all subjects.

### Pilot study to optimize MRI parameters

A pilot study (data not shown) determined the technical parameters for MRI imaging and the optimal times to perform MRI scans post allergen challenge. Two MRI scans were performed after the allergen challenge: an early scan after 15–30 min and a second scan at 45–60 min. The MRI data were reviewed by a radiologist and MRI parameters were defined for all subsequent scans in the main study (see Results). The optimal time to perform the MRI scan was found to be at 60 min post‐challenge when there were maximal inflammatory changes. However, at this time point the TNSS response was submaximal at 80% of the peak response which occurred at the earlier 15 min time point as we have previously described 13.

### Controlled trial of rhinitis therapies

**Figure 1:**
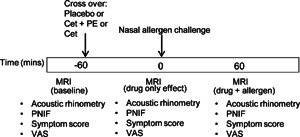
Study design with MRI assessments at baseline, 60 min after drug (pre‐challenge) and 60 min after allergen challenge. PNIF, peak nasal inspiratory flow; VAS, visual analog scale.

The study design is shown in Figure 1. This was a randomized, single‐blind, placebo‐controlled, double‐dummy, three period, crossover single oral dose study in patients with allergic rhinitis. Subjects were assessed at baseline with MRI scan, symptom scores, plus measurements of nasal patency. The same order of investigations were repeated at the different time points throughout the study. Subjects were then dosed according to the randomization code. Subjects were blindfolded before each dose administration. Active treatments and double dummy placebo were administered with 50 mL water and tablets swallowed under direct observation by the study nurse. The study medications were as follows: CONTACT 120 mg (pseudoephedrine hydrochloride). Zirtek 10 mg (cetirizine hydrochloride). There were matching placebo tablets for both Zirtek, and CONTACT 120.

The MRI scan and all measurements were repeated 60 min after drug administration and immediately before the allergen challenge procedure. Nasal allergen challenge was then performed (see below). The final MRI scan was repeated at 60 min post‐allergen challenge and all other measurements repeated. Each treatment period was separated by a wash‐out period of at least 7 days. This time period allowed the nasal mucosal process to return to a non‐symptomatic baseline.

### Nasal allergen challenge

Nasal allergen challenge was performed as previously described 1, 13 with Timothy grass pollen (Alk‐Abello, Denmark). Application of the allergen to the nasal mucosa was undertaken using a nasal pump spray (Dolphin nasal applicator, Valois 14, 15, 16. A total dose of 1 µg was given as 100 μL to each nostril (500 BU/mL to each nostril).

### Acoustic rhinometry and PNIF

Rhinometry was performed at screening pre‐challenge pre‐dose, and 60 min after challenge. Rhinometry assessments were made of nasal volume and minimum cross sectional area (ARMXCA) using an A1 acoustic rhinomter (GM Instruments, Kilwinning, UK). With a nosepiece inserted, subjects were asked to breathe in and hold their breath. The measurement was repeated three times. PNIF and maximum PNIF (PNIFMAX) were measured with a Portable Nasal Inspiratory Flow Meter (Clement Clark, Harlow, UK), with full exhalation beforehand. The PNIF was a short, sharp inspiratory action of approximately one second duration within an anesthetic mask tightly applied to the face. The measurement was repeated three times and the highest result recorded.

### nNO

nNO was measured using the NIOX analyser (Aerocrine, Stockholm, Sweden) with sample time of 40 s, with tidal breathing for approximately 5 s. The subject inhaled to total lung capacity over 2–3 s with the mouth open, then closed the mouth and held the breath as long as possible. Sample flow did not fall more than 1.5 mL/s below its normal value (around 5 mL/s). The procedure was repeated to obtain three readings within 10% of each other.

### Symptoms

Patient assessment of nasal patency was performed using VAS 0–100 mm. and TNSS completed by the subject. TNSS were recorded before and at frequent intervals after nasal allergen challenge. Nasal congestion, rhinorrhoea, sneezing, and nasal itch were scored from 0 to 3 (0 no, 1 mild, 2 moderate, 3 severe symptoms). The scores were then added up to give a final TNSS out of a maximum of 12.

### MRI measurements

MRI measurements were made using a Siemens 1.5T MRI scanner (Siemens Heathcare, Erlangen, Germany) with an average scan time for each subject approximately 15 min. Settings were 4–5 min for proton density (PD) and transverse relaxation time (T2) coronal views (4–5 min) with 40 slices, the dimensions were field of view (the size of the two dimensional spatial encoding area of the image; 160 × 143 mm^2^), matrix (384 × 205) and voxel size (0.7 × 0.4 × 0.3 mm^3^); time to equal (PD weighting = 43 ms; T2 weighting = 128 ms); time to repeat (6120 ms). Cross‐sectional airspace area (and thus also airspace volume) of the nasal passages was measured. Within subject, the cross‐sectional airspace area is directly proportional to the airspace volume per slice.

The primary endpoint was average airspace sectional area (METASV). Secondary MRI endpoints were: volume of fluid identified adjacent to the airspace; total mucosal surface area (TMSA); total nasal cavity volume (TNCV; a single value which could be applied to all time points per patient—either the single value derived following image registration, or the average of the values used for each time point per patient visit) and nasal tissue volume (NTV) derived from the nasal cavity volume less airspace and fluid volumes (the resulting volume included cartilage, bone, enclosed airspace and other factors and was only indicative of the tissue volume).

All MRI endpoints were considered in two ways: regularly defined intervals along the nasal passage, to allow standardized subject profiles to be plotted, and totals or averages along the entire length of the nasal passage to provide a simple subject‐level summary at each time point (additionally, the smallest cross‐sectional airspace area in any slice along the nasal passage and the location of this slice was determined). The maximum number of slices per subject was 21 so location specific MRI measurements were derived over 20 slices. MRI assessments of congestion included: total airspace volume (TASV), METASV, minimum cross‐sectional area (MIXA), TNCV, and TMSA.

### Statistics

This exploratory study was not formally powered, but was based on feasibility and exploratory statistics were used. Subjects were randomized with RANDALL. All statistical analyses were performed using SAS® on a windows or UNIX platform. Most displays were generated in the harmonization of Analysis and Reporting Program environment which uses SAS version 8.2 on a UNIX platform. Trellis plots were generated using S‐Plus version 7.0. Average air space sectional area was analyzed by mixed effect model, looking at change from baseline at pre‐ and post‐challenge. The model used fitted terms for baseline value, period, treatment, time and treatment–time interaction. The average baseline TNCV was explored as a covariate. The UNR correlation structure between time points was used. Subject was fitted as random variable. Point estimates and 95% confidence intervals were constructed using the appropriate variance term for the estimating the change from baseline at pre‐ and post‐challenge and the difference between pre‐ and post‐challenge for each treatment. The Kenward Roger correction was employed. Estimates of both within‐ and between‐subject variability were calculated for future studies, with 95% confidence intervals based on Satterthwaite's approximation. Similar models were used to analyse MIXA, TASV, and TMSA as well as normalized airspace volume (TNCVm). Similar models, but without adjusting for TNCV at baseline, were used to analyse total NTV (TNTV) and normalized nasal tissue. Acoustic rhinometry endpoints: nasal volume and MIXA, VAS, and PNIF changes from baseline were also analysed in a similar way to MRI endpoint without the adjustment for TNCV.

## Results

### Pharmacodynamic marker results

**Figure 2:**
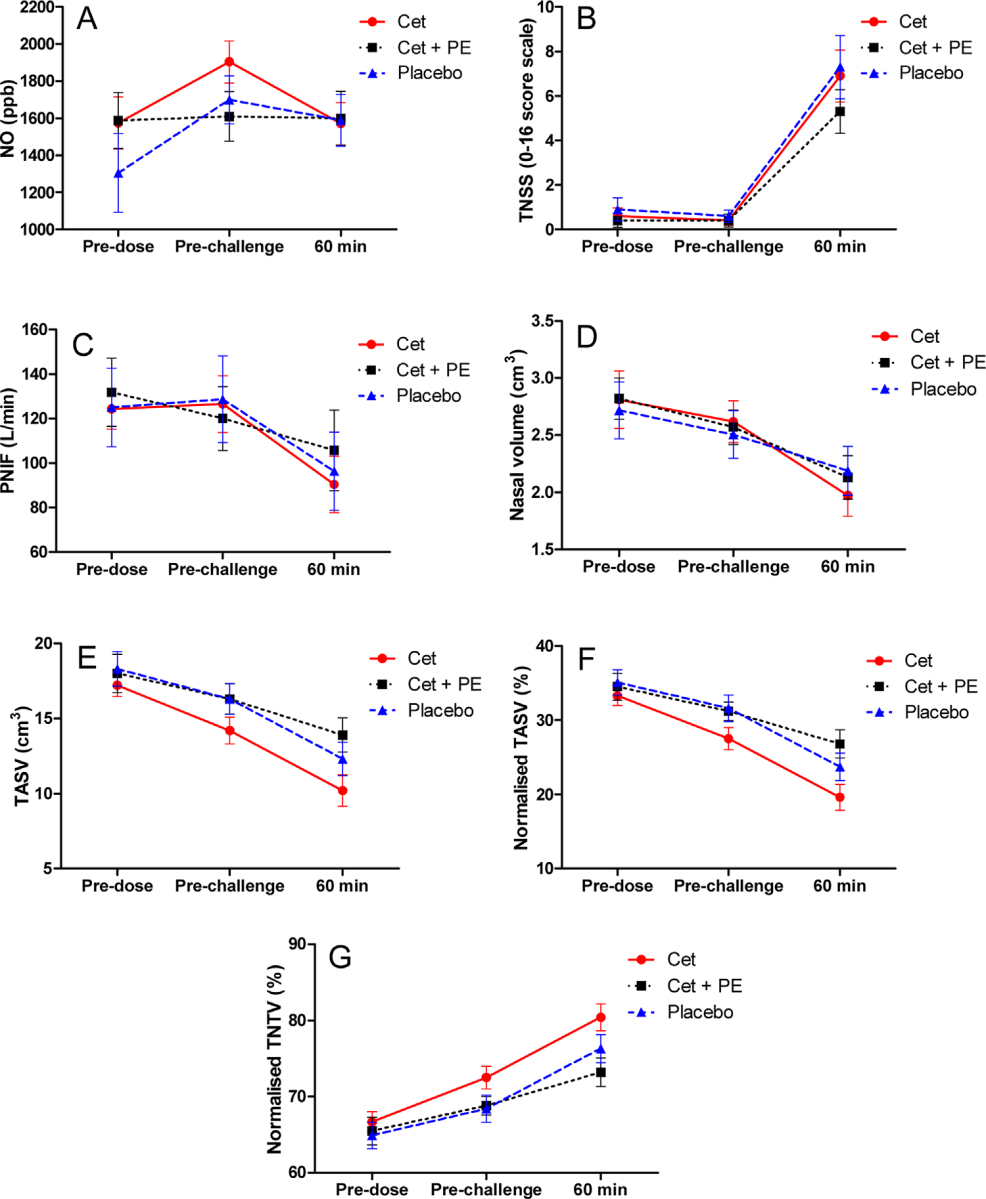
Panels for measurements pre‐dose and post‐nasal allergen challenge for the different treatment groups. (A) Nitric oxide (NO), (B) total nasal symptom score (TNSS), (C) peak nasal inspiratory flow (PNIF), (D) nasal volume, (E) total airspace volume (TASV), (F) normalized TASV, (G) normalized total nasal tissue volume (TNTV). Data are mean ± SD.

**Figure 3:**
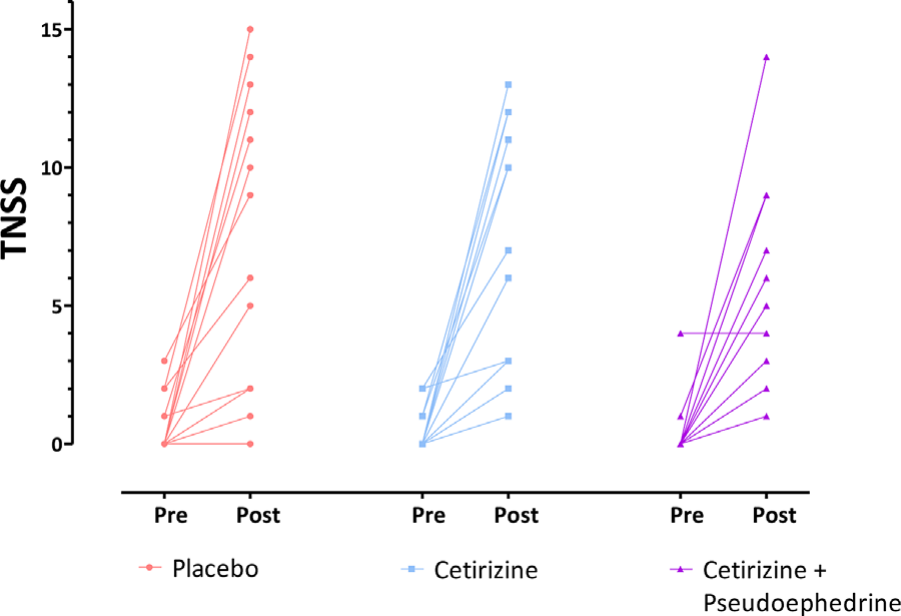
Total nasal symptom score (TNSS) pre‐allergen challenge (after dosing) and 60 min post‐nasal allergen challenge for individual subjects with the different treatments.

Allergen challenge produced a significant change across all measurements (Fig. 2 and Table 1). The individual data for TNSS pre‐ and post‐allergen challenge for the different treatment periods are shown in Figure 3. The mean change and changes from baseline for TNSS was Δ6.55 (95%CI 4.16, 8.94; *P *< 0.001), PNIF was Δ−28.77 (95%CI −46.58, −10.96; *P *< 0.002), and VAS was Δ26.67 (95%CI 12.20, −4114; *P *< 0.001). For the MRI measurements, the change from baseline values were; TASV Δ−5.37 (95%CI −7.35, −3.38; *P *< 0.001), NTV Δ5.47 (95%CI 3.25, 7.70; *P *< 0.001), TMSA Δ−37.74 (95%CI −61.40, −14.08; *P *< 0.003). TMSA also was also reduced by allergen, mean difference Δ−37.74 (95%CI −61.40, −14.08; *P *< 0.03).

**Table 1:** Changes induced by allergen challenge in the presence of treatment can be detected by most endpoints (LS means (95%CI) of treatment estimate of change from pre‐challenge)

Treatment	Estimate	Lower	Upper	*P*
NSSC				
Cet	1.72	0.96	2.48	0.001
Cet + PE	1.31	0.53	2.08	0.002
Placebo	1.41	0.64	2.18	0.001
PNIF				
Cet	−36.29	−59.14	−13.45	0.003
Cet + PE	−13.31	−35.27	8.65	0.227
Placebo	−33.48	−55.43	−11.54	0.004
TASV				
Cet	−4.29	−6.32	−2.27	0.001
Cet + PE	−2.28	−4.23	−0.32	0.024
Placebo	−3.76	−5.80	−1.73	0.001
TNSS				
Cet	6.40	3.97	8.84	0.001
Cet + PE	4.77	2.32	7.21	0.001
Placebo	6.83	4.38	9.29	0.001
VAS				
Cet	27.08	13.64	40.52	0.001
Cet + PE	20.17	6.73	33.61	0.004
Placebo	26.76	12.79	40.72	0.001

Cet, cetirizine; PE, pseudoephedrine; NSSC, nasal symptom score for congestion; PNIF, peak nasal inspiratory flow; TASV, total airspace volume; TNSS, total nasal symptom score; VAS, visual analog score.

**Figure 4:**
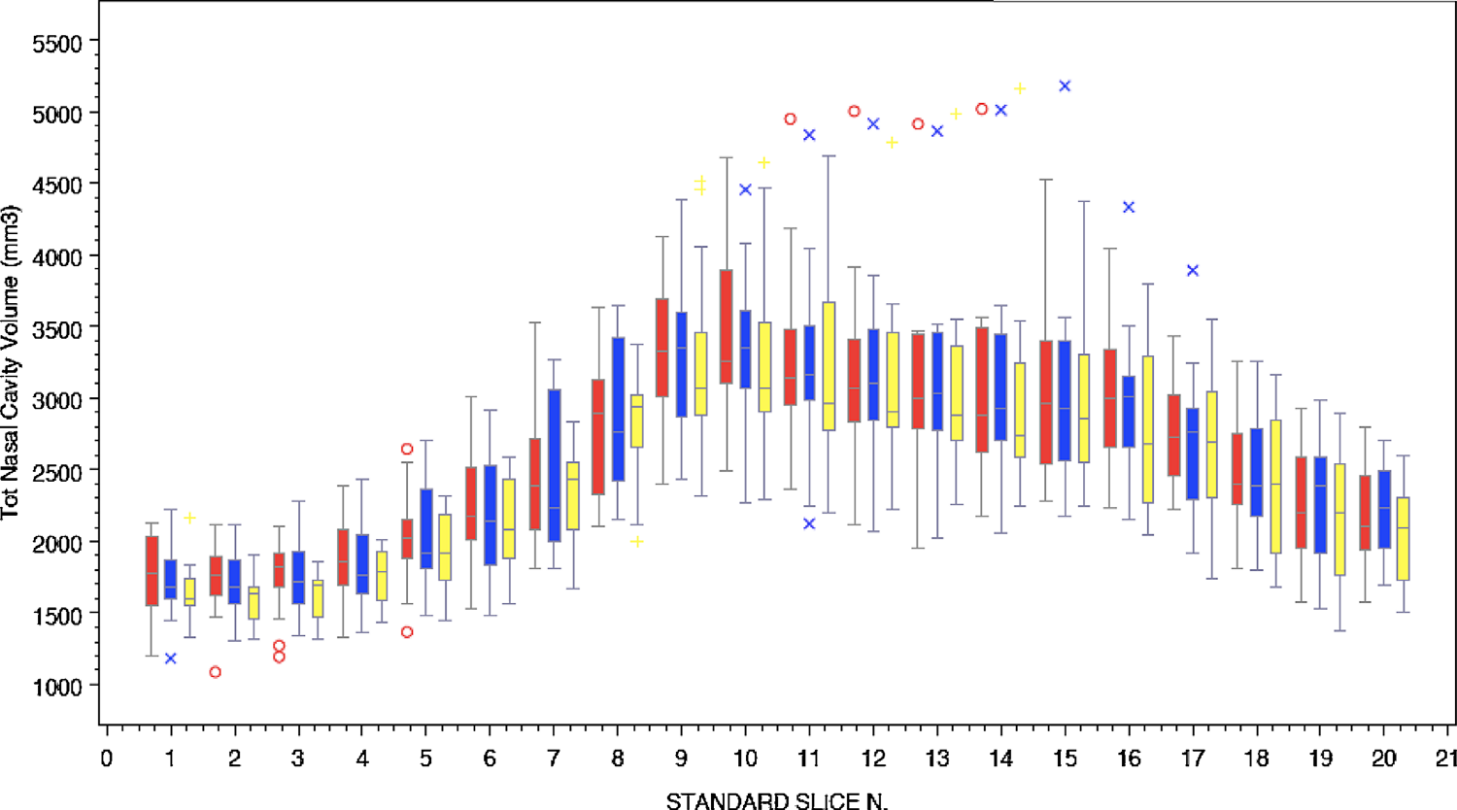
Box plot of MRI versus standardized slice number with total airspace volume (TASV) mm for placebo group (10 yellow, blue 60, and red 120 min relative to dosing with allergen). Bars represent median, upper and lower quartiles, whiskers represent 5–95% percentiles. Outliers are represented as symbols o and x.

**Figure 5:**
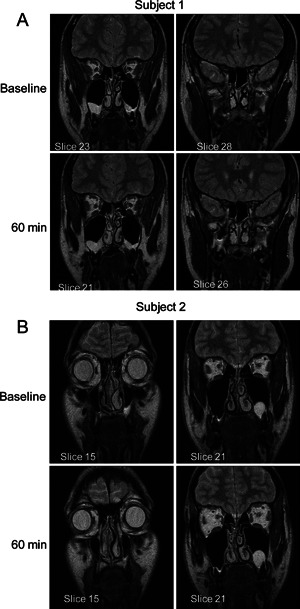
(A) MRI at baseline and at 60 min following challenge; subject 1 shows decreased airspace in both nostrils, the changes are representative for all but one of the subjects where the baseline scan showed movement artifact; (B) MRI at baseline and at 60 min following challenge; subject 2 shows unilateral changes with decreased airspace in right nostril, increased airspace in left nostril.

The variability in the intra‐subject response and the different methodologies employed were NSSC 1.08 (95%CI 0.85, 1.53); PNIF 38.32 (95%CI 29.68, 54.11); TASV 2.98 (95%CI 2.30, 4.21); TNSS 3.63 (95%CI 2.82, 5.09); VAS 22.07 (95%CI 16.98, 31.53). The lowest intra‐subject variability was seen with MRI measurement of TASV. The MRI profile with slice is shown in Figure 4 and typical MRI images pre‐ and post‐allergen challenge shown in Figure 5. The changes in volume in a typical subject are clearly seen and a unilateral predominance in some subjects (Fig. 5B).

There is some indication of correlation between the MRI and acoustic rhinometry volume (ARV), and some degree of negative correlation between MRI and VAS score. A change of one in the TNSS score on average corresponded with MRI volume of −0.57 cm^3^, with a range of −0.78 to −0.37. The correlation data can be viewed in the supplemental data online (Figures S1 and S2).

There was a trend towards difference between treatment and placebo although this did not achieve significance because of small numbers (Fig. 2; *P *= 0.32); across allergen challenge the differences between time‐points were highly significant (*P *< 0.001). There was no evidence of an interaction between time and treatment (*P *> 0.1).

Overall across all treatments (Fig. 2 and Table 2) the airspace sectional area tended to decrease after challenge. The drop was less pronounced for the combination treatment Cet plus PE, although not significantly different from placebo. Similar results were obtained when analysing airspace volume. The graphical analysis shows the trend for a treatment separation that did not reach statistical significance but illustrates the lower variability with MRI than with other methods (Table 2).

**Table 2:** Effect of allergen challenge on MRI endpoints. LS means (95%CI) of treatment comparison for change from pre‐challenge

Treatment	Ref	Test‐ref	Lower	Upper	*P*
NSSC					
Cet	Cet + PE	0.41	−0.46	1.27	0.34
Cet + PE	Placebo	0.31	−0.56	1.17	0.47
Placebo	Placebo	−0.10	−1.00	0.79	0.82
PNIF					
Cet	Cet + PE	−22.98	−53.80	7.84	0.14
Cet + PE	Placebo	−2.81	−33.50	27.88	0.85
Placebo	Placebo	20.17	−9.98	50.33	0.18
TASV					
Cet	Cet + PE	−2.02	−4.43	0.39	0.10
Cet + PE	Placebo	−0.53	−2.99	1.92	0.66
Placebo	Placebo	1.49	−0.92	3.90	0.21

Cet, cetirizine; PE, pseudoephedrine; NSSC, nasal symptom score for congestion; PNIF, peak nasal inspiratory flow; TASV, total airspace volume; TNSS, total nasal symptom score; VAS, visual analog score.

### VAS, TNSS, and PNIF

There was no evidence of treatment difference or treatment time interaction (all *P *> 0.5) and, overall across all treatments, the VAS increased after challenge (*P *< 0.001; Fig. 2). The increase was less pronounced for the combination treatment Cet plus PE, although not significantly different from placebo. The TNSS results were similar to those of VAS (Fig. 2). PNIF showed a trend to fall after allergen challenge and across all treatment groups (Fig. 2).

### Acoustic rhinometry

There was no evidence of difference between treatments or an interaction between time and treatment (*P*> 0.1; Table 2). There was evidence of difference between time points (*P* < 0.001; Fig. 2 and Table 1). The trend was similar to the one observed when analysing the MRI METASV.

### nNO

There were no significant changes in nNO across the allergen challenge procedure (Fig. 2). On placebo pre‐dose values were 1304 ± 762 ppb; post‐dose 1699 ± 485; post‐challenge 1509 ± 522; Cet treated group pre‐dose value was 1587 ± 565; post‐dose 1609 ± 500; 1599 ± 523 post‐challenge. In the combination group, the values were pre‐dose 1574 ± 589; post‐dose 1904 ± 500; post‐challenge 1570 ± 430. The data showed no significant change (Fig. 2) following allergen challenge.

### Safety

There were no serious adverse events reported. The MRI scans and procedure was well tolerated. Adverse events were mostly related to the allergen challenge and were mild in nature and self‐limiting.

## Discussion

This is the first study in man to evaluate nasal allergen responses using MRI imaging before and after nasal allergen challenge. Animal studies have reported the effect of nasal challenge in response to histamine and methacholine on MRI images using gadolinium enhancement to localize edema formation 17, 18, 19, 20, 21. Clinically, MRI has mainly been used for the evaluation of the para‐nasal sinuses and may replace CT for the evaluation of pathological mucosal abnormalities 22, 23. A recent report describes the use of MRI to evaluate the inflammation produced by segmental bronchial allergen challenge 24.

Nasal allergen challenge produced a highly significant change in all the measured variables including all MRI endpoints. The timing and magnitude of the changes seen after allergen challenge seen in this study for symptom‐based scores and measurements of nasal patency were similar to that reported in a recent review, which included a dose‐response analysis to nasal administration of nasal allergen 1. Therefore, the findings in our study that MRI endpoints, particularly TASV, were more sensitive and less variable than the conventional measurements of symptom scores and nasal patency would suggest that MRI may prove a useful and objective method for assessment of the inflammatory response post‐allergen challenge. A negative change is indicative of smaller free nasal airspace. A drop in airspace volume was expected after allergen challenge. A smaller negative change from baseline would represent a lower reaction from the nasal tissues to the allergen challenge and that would then be reflected in wider free nasal airspace.

The clearest separation of the different treatments was seen in the MRI measures of the volume of the respiratory space and the soft tissue, normalized total nasal tissue volume (TNTV; Fig. 2). An advantage of MRI is the ability to measure mucosal inflammatory changes and edema as well as volume changes directly.

In some instances, the changes in volume were localized to one side, as shown in Figure 5B. This is consistent with an asymmetry of nasal volume changes post‐allergen challenge, (with greater changes on the non‐patent side) which was related to the endogenous nasal cycling rhythm 25. Measures of cross‐ sectional area have been compared between MRI and acoustic rhinometry 26, although the extent of the correlation has been reported to depend on the state of congestion which limits its value in challenge studies 27. The lack of correlation between the different variables illustrates the difficulty in utilization of symptom‐based scores to assess the effects of nasal challenge 3, 4, 28 and it should be pointed out that one method for assessment is not adequate for allergic rhinitis assessment and that multiple methods should be used. There was a weak correlation between the MRI and ARV, and a weak negative correlation between MRI and VAS (supplemental data). A lack of correlation with symptom VAS score and cross‐sectional area measurements by acoustic rhinometry was also seen in a histamine challenge 29. No difference was seen in nNO data. The baseline values are in the range reported by Kharitonov 30 and somewhat higher than the values in normal controls reported in other studies 31. However, the effects of nasal allergen challenge are varied with decreases in nNO reported 30, or no change 32, probably dependent on the degree of sinus obstruction.

The lack of a clear difference between drug treatments and placebo may have been due to the single dose administration of active drugs and restricted time‐point analysis. The study was not powered to show statistically significant differences between drug treatments and placebo. There was a clear trend that suggested a positive treatment effect after combination therapy (Cet+PE); the data showed a reduction in symptom scores, improvement in nasal patency, and MRI end points (Fig. 2), although statistical significance was not achieved perhaps because of the small numbers involved (*n* = 14). These changes may result from the effect of PE in reducing vascular congestion and edema. Other studies have also demonstrated that the combination of anti‐histamines with vasoconstrictor agents increases efficacy in the treatment of allergic rhinitis 7, 12.

By contrast, a single dose of Cet failed to abrogate the effects of allergen challenge and post‐allergen measurements were no different from placebo. A similar lack of effect of Cet on nasal patency as recorded by acoustic rhinometry (MIXA) has also been reported 29. Antihistamines have minimal effects on nasal congestion, which remains one of the less well‐treated symptoms of allergic rhinitis. After single dosing with anti‐histamines, effects are typically seen on cutaneous topical challenge wheal and flare responses up to 24 h after a single dose 33. More often effects of anti‐ histamines have been examined after chronic dosing. The effect of anti‐ histamines is greatest on the itching and sneezing symptoms scores 28, typically measured at steady state. However, in a study comparing skin and nasal responses of five anti‐ histamines, inhibitory effects were seen after single dosing 11. Additionally, single dose studies with anti‐histamines using the Vienna Chamber 34 showed significant reductions in symptoms score of approximately 30% within 1 h post‐dosing, but with much larger sample sizes (*n* = 65). Evidence of synergy of the effects has been previously reported for the combination of PE and Cet in chronic dosing studies 7, 8. Differences between single dose and multiple dose effects are also seen in other studies with theophylline 35 and with corticosteroids, where acute and chronic effects seen with inhaled corticosteroids differ when administered immediately before challenge corticosteroids have a small effect on early phase symptoms and inhibit predominantly the late phase TH2 mediated responses (IL‐4, ‐5, ‐13) 15, 16. In contrast, multiple dosing with corticosteroids inhibits symptoms in the early phase (approximately 30% 15, 36 and attenuates predominantly the late phase production of TH2 cytokines in response to allergen challenge 15, 36. Changes in the airway calibre measured with acoustic rhinometry with corticosteroids, showed a trend but did not reach statistitical significance again in contrast to effects of inhaled corticosteroid on symptom scores 37. The variability of rhinometric measurements varies from 10% to 30% and is beset by intrinsic problems of the non‐linear relationship between flow and volume 4. Additionally, expressing the rhinometric measurements as a minimal cross sectional area to depict resistance is complicated by non‐uniform flow and change between laminar and turbulent flow 9, 38.

This is the first reported study of the use of MRI where this technique has been used to quantify the inflammatory changes following nasal allergen challenge. MRI endpoints have been compared directly with conventional assessments, such as symptom scores and measurements of nasal patency, although subjective measures might be variable, they are a good indication to how the participant perceives their allergic rhinitis symptoms, and currently the FDA recognizes TNSS as the only parameter in evaluating new medications, while still considering others. The major MRI limitations remain cost, imaging time, and the potential for claustrophobia. MRI procedures were well tolerated in this small study despite the need to keep perfectly still following allergen challenge, and the acquisition times prevent multiple frequent measurements. MRI provides an objective method for the assessment of the response to nasal challenge, which merits further study in studies with chronic dosing and novel agents. MRI provides novel insights into the anatomical changes in response to allergen and provides a new method for the assessment of nasal patency, vascular congestion, and inflammation.

## Conflict of Interest

BRL has received research funding from AstraZeneca, Chiesi, Daiichi‐Sankyo, DSP‐Sumitomo, GlaxoSmithKline, Pfizer. GS has received research grants from GlaxoSmithKline, ALK, honoraria for articles, consulting, lectures/chairing and/or advisory boards from AstraZeneca, Brittania Pharmaceuticals, Capnia, Church & Dwight, Circassia, GlaxoSmithKline, Groupo Uriach, Meda, Merck, MSD, Ono Pharmaceuticals, Oxford Therapeutics, Sanofi‐Aventis, Shionogi, UCB and travel funding from Bayer, GlaxoSmithKline. CRJ has no declared competing interests. PJB has served on Scientific Advisory Boards of AstraZeneca, Boehringer‐Ingelheim, Chiesi, Daiichi‐Sankyo, GlaxoSmithKline, Novartis, Nycomed, Pfizer, Teva and UCB and has received research funding from Aquinox Pharmaceuticals, AstraZeneca, Boehringer‐Ingelheim, Chiesi, Daiichi‐Sankyo, GlaxoSmithKline, Novartis, Nycomed, Pfizer and Prosonix. He is also a cofounder of RespiVert (now part of Johnson & Johnson), which has discovered novel inhaled anti‐inflammatory treatments for asthma and COPD.

## Supporting information

Additional supporting information may be found in the online version of this article at the publisher's web‐site.

**Figure S1**. Correlation data from MRI for total airspace volume (TASV) versus total nasal symptom score (TNSS). The changes between pre‐challenge and post‐challenge are plotted. Parameter estimates; 95% confidence limits; slope −0.57 (−0.78, −0.37).
**Figure S2**. Trellis plot of MRI derived measurement versus acoustic rhinometry data, all time points all data. The average airspace volume is directly proportional to the total airspace volume. The black box outlines the MRI analyses; total airspace volume (TASV), average airspace sectional area (METASV), minimum cross‐sectional area (MIXA), total mucosal surface area (TMSA) and total nasal cavity volume (TNCV). Data for the visual analog scale score (VASCR), acoustic rhinometry distance of minimal cross‐sectional area (ARDX), acoustic rhinometry volume (ARV), acoustic rhinometry cross‐sectional area (ARX), Maximum peak nasal inspiratory flow (PNIFMAX). There are indications of correlation between the MRI assessments and more conventional endpoints and suggestions of greater correlations between VASCR and ARV (cells with red box outline). Symbols: + placebo, o cetirizine, Δ cetirizine + pseudoephedrine; red 60 min post‐challenge, green post‐dose, blue pre‐challenge.

**Table S1**. Demographic characteristics of seasonal rhinitis subjects studied. Data are mean ± SD.
