# Supplementary figures and images for: Using magnetic resonance imaging to quantify the inflammatory response following allergen challenge in allergic rhinitis
Source: Immun Inflamm Dis. 2015 Sep 17;3(4):445–54. doi: 10.1002/iid3.86 (PMC4693719; doi:10.1002/iid3.86)

## Slide 1
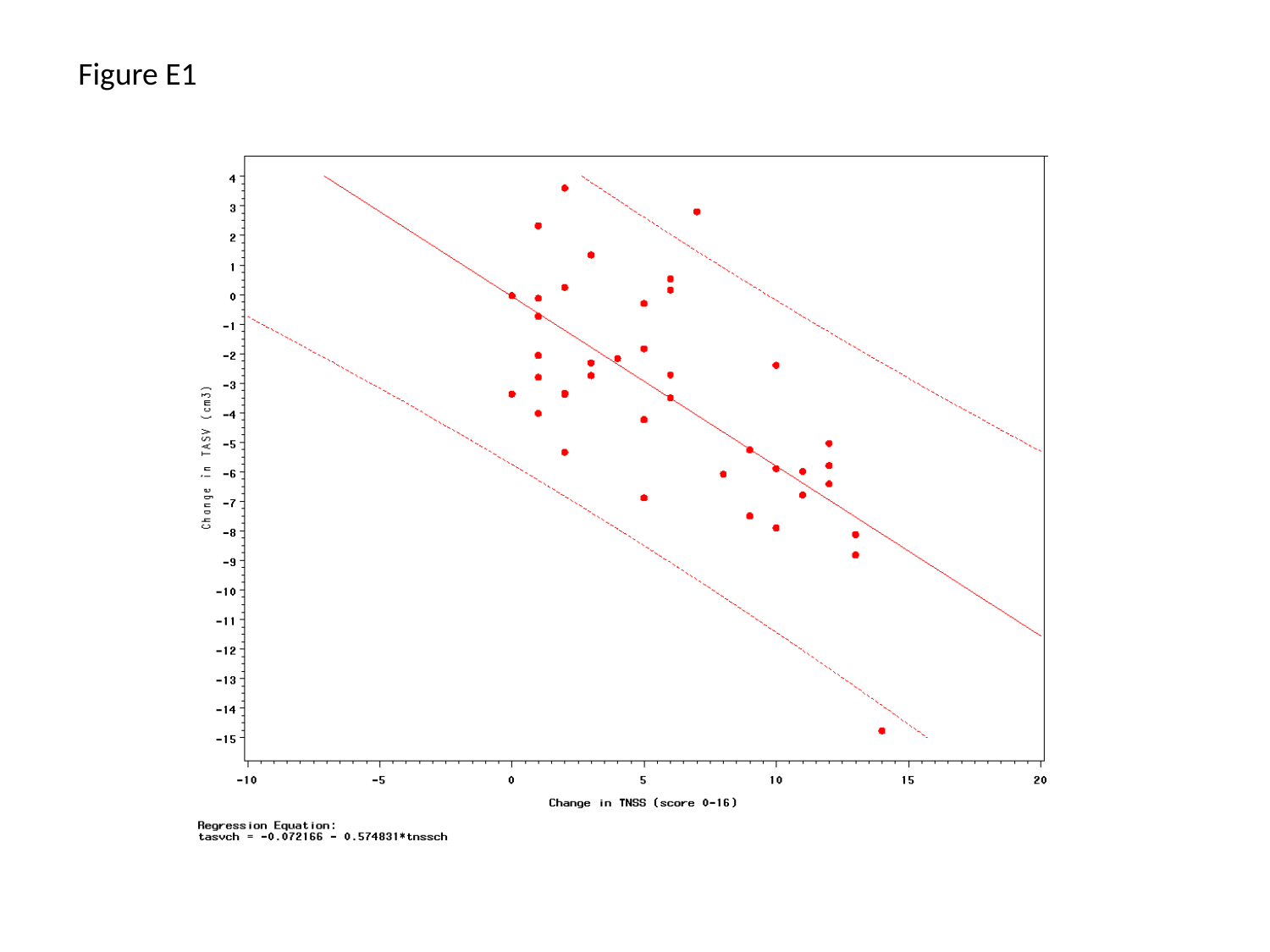

Figure E1

## Slide 2
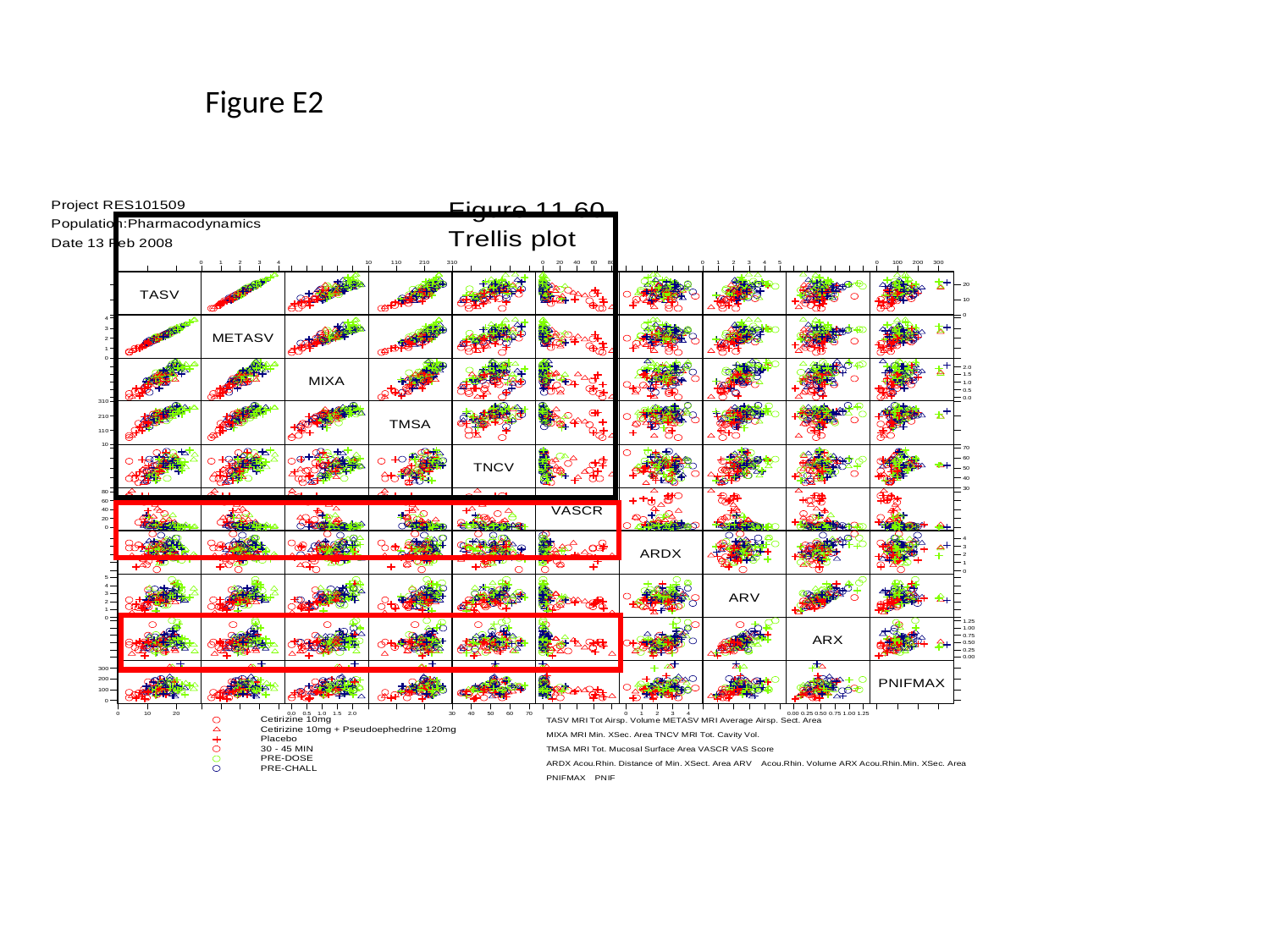

Figure E2

Supplement: Supplementary file 1 — Figure S1. Correlation data from MRI for total airspace volume (TASV) versus total nasal symptom score (TNSS). The changes between pre‐challenge and post‐challenge are plotted. Parameter estimates; 95% confidence limits; slope −0.57 (−0.78, −0.37). Figure S2. Trellis plot of MRI derived measurement versus acoustic rhinometry data, all time points all data. The average airspace volume is directly proportional to the total airspace volume. The black box outlines the MRI analyses; total airspace volume (TASV), average airspace sectional area (METASV), minimum cross‐sectional area (MIXA), total mucosal surface area (TMSA) and total nasal cavity volume (TNCV). Data for the visual analog scale score (VASCR), acoustic rhinometry distance of minimal cross‐sectional area (ARDX), acoustic rhinometry volume (ARV), acoustic rhinometry cross‐sectional area (ARX), Maximum peak nasal inspiratory flow (PNIFMAX). There are indications of correlation between the MRI assessments and more conventional endpoints and suggestions of greater correlations between VASCR and ARV (cells with red box outline). Symbols: + placebo, o cetirizine, Δ cetirizine + pseudoephedrine; red 60 min post‐challenge, green post‐dose, blue pre‐challenge. [file IID3-3-445-s001.pptx]
