# Supplementary material for: Using magnetic resonance imaging to quantify the inflammatory response following allergen challenge in allergic rhinitis
Source: Immun Inflamm Dis. 2015 Sep 17;3(4):445–54. doi: 10.1002/iid3.86 (PMC4693719; doi:10.1002/iid3.86)
Supplement: Supplementary file 2 — Table S1. Demographic characteristics of seasonal rhinitis subjects studied. Data are mean ± SD. [file IID3-3-445-s002.docx]

**Table EI.** Demographic characteristics of seasonal rhinitis subjects studied. Data are mean ± SD.

| **Parameter** | **Total (n=14)** |
| --- | --- |
| Age (range) | 31(21-48) |
| Sex (M;F) | 12;2 |
| FeNO (ppb) | 1305 ± 763 |
| VAS (0-100mm) | 3.4 ± 5.77 |
| TNSS | 0.9 ± 1.94 |
| PNIF(L/min) | **125 ± 66** |
| Acoustic rhinometry distance cm2minXsec area | **2.80 ± 0.59** |

Abbreviations – FeNO: fractional exhaled nitric oxide; PNIF: peak nasal inspiratory flow; TNSS: total nasal symptom score; VAS: visual analogue score
